# Aberrant DNA methylation of GATA binding protein 3 (GATA3), interleukin-4 (IL-4), and transforming growth factor-β (TGF-β) promoters in Behcet's disease

**DOI:** 10.18632/oncotarget.19500

**Published:** 2017-07-22

**Authors:** Yunyun Zhu, Yiguo Qiu, Hongsong Yu, Shenglan Yi, Wencheng Su, Aize Kijlstra, Peizeng Yang

**Affiliations:** ^1^ The First Affiliated Hospital of Chongqing Medical University, Chongqing Key Laboratory of Ophthalmology and Chongqing Eye Institute, Chongqing, China; ^2^ University Eye Clinic Maastricht, Maastricht, The Netherlands

**Keywords:** DNA methylation, Behcet’s disease, CD4^+^ T cell

## Abstract

The pathogenesis of Behcet's disease (BD) remains poorly understood. The purpose of this study was to investigate whether an aberrant DNA methylation of transcriptional and inflammatory factors, including *TBX21, GATA3, RORγt, FOXP3, IFN-γ, IL-4, IL-17A* and *TGF-β*, in CD4^+^T confers risk to BD. We found that the promoter methylation level of *GATA3, IL-4* and *TGF-β* was significantly up-regulated in active BD patients and negatively correlated with the corresponding mRNA expression. The mRNA expression of *GATA3* and *TGF-β* was markedly down-regulated in active BD patients compared to healthy individuals. Treatment with corticosteroids and cyclosporine (CsA) resulted in a decrease of the methylation level of *GATA3* and *TGF-β* in inactive BD patients. Our results suggest that an aberrant DNA methylation of *GATA3* and *TGF-β* is associated with their mRNA expression and participates in the pathogenesis of BD.

## INTRODUCTION

Behcet's disease (BD) is an autoimmune-mediated multisystemic inflammatory disease featured by diverse clinical manifestations including recurrent uveitis, oral and genital ulceration, and multiform skin lesions. It is prevalent in the region of the ancient Silk Route, such as Japan, China and Turkey [1, 2]. Although the pathogenesis of BD is still not clear, numerous studies have suggested that environmental factors, infection triggers, abnormal autoimmune regulation and genetic susceptibility are closely associated with BD [3]. Previous studies have demonstrated a crucial role for T lymphocytes in the pathogenesis of autoimmune diseases, including BD. Early studies reported that hyperactive T helper (Th) type 1 and Th17 immune responses were related to the development of BD [4, 5].

T helper (Th) type 1, Th2, Th17 and regulatory T cell (Treg) are the major subpopulations of CD4^+^T cells. *TBX21* (Th1)*, GATA3* (Th2)*, RORγt* (Th17) and *FOXP3* (Treg) are the primary subset-specific transcription factors of these CD4^+^T cell subsets and can regulate T cell differentiation. An earlier study found that the ratio of Th1/Th17 cells was decreased in BD patients with uveitis [5], and another study reported an increase in the ratios of *RORC*/*FOXP3* and *TBX21*/*GATA3* in neuro-Behçet's disease, suggesting a dysregulation of Th1, Th2, Th17 and Tregs in this disease [6]. *Interferon-γ, IL-4, IL-17* and *TGF-β* are the central inflammatory cytokines of Th1, Th2, Th17 and Treg cells, respectively. The expression of lineage-specific transcription factors is also crucial in regulating the production of T helper profile-associated cytokines. A previous study from our team showed that the mRNA expression of *IL-17* and *IFN-γ* was significantly increased in active BD patients [4], and another study showed that cyclosporine A (CsA) can markedly inhibit the production of both *IL-17* and *IFN-γ* in BD patients [7]. Earlier studies also showed that the cytokine production of *IL-4* was higher in peripheral blood mononuclear cells (PBMCs) and serum from patients with BD [8, 9].

DNA methylation refers to enzymatic addition of a methyl group to the fifth carbon of cytosine at CpG motifs, which is one of the crucial epigenetic mechanisms that can regulate the gene expression without changing the DNA sequence [10–12]. DNA hypomethylation is closely related to transcriptional activation, while DNA hypermethylation is related to transcriptional silencing [13]. In recent years, a growing number of studies recognized the importance of DNA methylation in the immune response, especially in the cytokines produced during T cell differentiation [13–15]. Furthermore, various studies have shown that an abnormal DNA methylation may play an important role in the occurrence and development of many tumors [16–18]. In addition, accumulating evidence is available to show that aberrant DNA methylation of CD4^+^T cells may play a key role in the pathogenesis of several immune mediated disorders [19–21]. Considering the crucial role of DNA methylation and CD4^+^T cells in the pathogenesis of inflammatory disease, we decided to investigate whether DNA methylation of the major transcription factors and cytokines of CD4^+^T cells had an impact on the development of BD. Our results suggest that hypermethylation of *GATA3, IL-4* and *TGF-β* confers risk to BD.

## RESULTS

### Increased methylation level of the *GATA3, IL-4* and *TGF-β* promoters was observed in CD4^+^T cells from active BD patients

**Figure 1:**
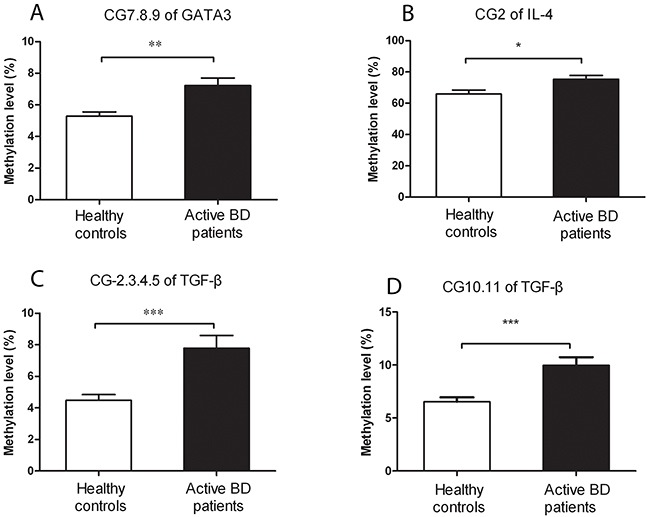
A higher methylation level of *GATA3, IL-4* and *TGF-β* is detected in CD4^+^ T cells from active BD patients (n=16) when compared healthy controls (n=18) Methylation levels of the CpG-7. 8.9 unit in *GATA3* **(A)**, CpG-2 in *IL-4* **(B)**, as well as CpG-2.3.4.5 **(C)** and CpG-10.11 **(D)** in *TGF-β* were all significantly up-regulated in BD patients compared to that in healthy controls. Data represent mean ± SEM. * P < 0.05, ** P < 0.01, *** P < 0.001.

To investigate whether the methylation level of master transcription factors (*TBX21, GATA3, RORγt* and *FOXP3*) and inflammatory factors (*IFN-γ, IL-4, IL-17A* and *TGF-β*) in CD4^+^T cells is associated with BD, we determined the promoter methylation level in active BD patients and healthy individuals. Because of the limitation of the MassARRAY system, we can only detect target sequences with a length between 100 to 500 base pairs. Detailed information of the target genes tested in this study is shown in Table 1. We were able to detect 8 CpG sites in the *GATA3* promoter whereby the methylation level of the CG-7.8.9 unit was found to be remarkably higher in active BD patients than that in normal subjects (P=0.001, Table 2, Figure 1A). The methylation level of the other CpG sites was not significantly different between the two groups (Table 2). In the *IL-4* promoter, we were able to detect 2 CpG sites and found a hypermethylation of the CG-2 site in active BD patients as compared to healthy subjects (P=0.012, Table 2, Figure 1B). A total of 9 CpG sites were detectable in the *TGF-β* promoter and the methylation level of CG-2.3.4.5 and CG-10.11 units was significantly up-regulated in active BD patients when compared with controls (P=4.65×10^−4^, P=2.85×10^−4^, respectively. Table 2, Figure 1C and 1D). The methylation levels of all CpG sites detected in this study are shown in Table 2.

**Table 1:** Primer sequence of target genes for amplifying bisulfite-treated DNA

Gene	Chr	Location	Primer sequence	Target Length	CpGs	Tm
***TBX21***	17	−593∼-411	forward:ggaagagagGTTTTTGAGTGTTAGGAGAATGTTTA reverse:taatacgactcactatagggagaaggctAAAATCTTACTTTCTAAAAATATCAACTCC	182	3	56
***RORγt***	1	−1228∼-1015	forward:ggaagagagGGGAGATTTTGGGAGTTATTTAAGA reverse:aatacgactcactatagggagaaggctAAACAACAAAACAAAAATCAACCAT	214	1	56
***GATA3***	10	−2316∼-2167	forward:aggaagagagTTTTTTTTGGTAGTATTGTTTTGGG reverse:cagtaatacgactcactatagggagaaggctATCCTTTAAACCACTACATCCCCTA	149	8	56
***FOXP3***	X	−307∼+86	forward:aggaagagagGATATTTTTTATTTTTGTGGTGAGGG reverse:cagtaatacgactcactatagggagaaggctCCTCCAATAAAACCCACATCTAATA	393	3	56
***IFN-γ***	12	−348∼+101	forward:aggaagagagAAGATTAGTTAAGTTTTTTGGATTTGATT reverse:cagtaatacgactcactatagggagaaggctCTACCTACAAAAAATAACAACCTATCA	297	1	56
***IL-4***	5	−182∼+182	forward:aggaagagagGTGTTGATTGGTTTTAAGTGATTGA reverse:cagtaatacgactcactatagggagaaggctAAACATCACCAAAACATCTAAAAAAA	364	2	56
***IL-17A***	6	−195∼+86	forward:aggaagagagGAGATTTTTTTATGATTTTATTGGGG reverse:cagtaatacgactcactatagggagaaggctTCCAAAAATACTATCTAATCCAAATCAAC	281	1	56
***TGF-β***	19	−701∼-495	forward:aggaagagagGTAGTTTGAGGTTTTAGAGTTTGAGA reverse:cagtaatacgactcactatagggagaaggctAAAATCCCCAAATCCTACCTCC	206	10	56

**Table 2:** Methylation levels of *TBX21, GATA3, RORγt, FOXP3, IFN-γ, IL-4, IL-17A* and *TGF-β* promoter in CD4^+^ T cell from BD patients versus normal controls

Gene	CG sites	BD methylation level (%,mean ± SD)	CN methylation level (%,mean ± SD)	p value
	CG2	63.8±3.6	62.5±7.7	0.53
***TBX21***	CG3	7.3±1.1	7.9±1.7	0.30
	CG4	8.8±1.6	9.4±2.0	0.61
	CG1	2.8±1.8	3.1±2.1	0.80
	CG3.4	6.5±1.6	5.8±1.9	0.43
***GATA3***	CG5	3.9±0.01	3.2±0.02	0.33
	CG6	4.7±3.3	2.9±1.9	0.13
	CG7.8.9	7.2±1.9	5.3±1.2	0.001**
***RORγt***	CG5	67.1±7.1	69.5±6.7	0.34
	CG2	36.7±14.3	34.1±10.1	0.30
***FOXP3***	CG3	38.6±15.9	35.8±6.3	0.86
	CG6	70.5±11.7	68.1±13.7	0.23
***IFN-γ***	CG3	68.1±11.6	66.2±10.8	0.64
***IL-4***	CG2	75.4±9.3	65.9±10.9	0.012*
	CG5	81.0±6.3	78.3±6.5	0.22
***IL-17A***	CG1	82.3±10.8	82.2±4.7	0.98
	CG2.3.4.5	7.8±3.2	4.5±1.5	4.65×10^−4^***
***TGF-β***	CG6	4.5±5.2	2.9±2.4	0.70
	CG10.11	10.0±3.1	6.5±1.7	2.85×10^−4^***
	CG13.14	2.3±2.2	1.9±1.7	0.65

BD patients (n=16, M: F=14:2); Age (year: mean±SD): 34.63 ± 6.98.

Normal controls (n=18, M: F=14:4); Age (year: mean±SD): 37.61± 13.63.

* P < 0.05, ** P < 0.01, *** P < 0.001.

### Decreased mRNA expression of *GATA3* and *TGF-β* was detected in CD4^+^T cells from active BD patients

**Figure 2:**
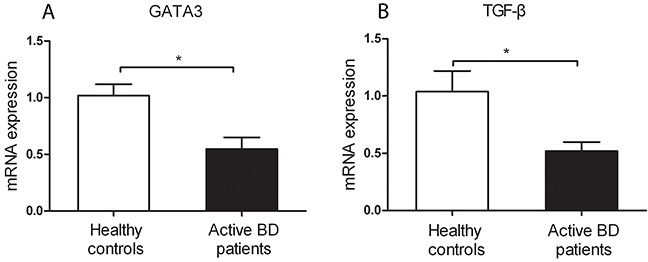
Decreased mRNA expression levels of *GATA3* (**A**) and *TGF-β* (**B**) were found in CD4^+^ T cell from active BD patients compared to healthy controls. BD patients: n=5; Normal controls n= 5. Data represent mean ± SEM. * P < 0.05.

**Figure 3:**
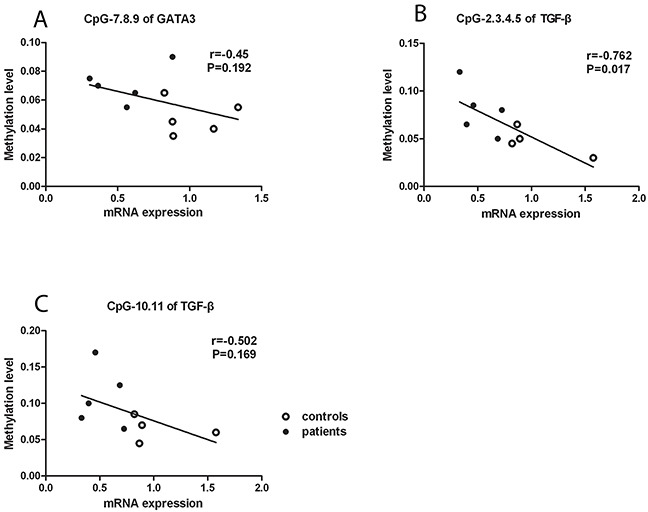
Negative correlation between DNA methylation and mRNA expression The DNA methylation level of the CpG-7. 8.9 **(A)** unit in *GATA3* as well as CpG-2.3.4.5 **(B)** and CpG-10.11 **(C)** units in *TGF-β* were negatively related to their mRNA expression.

To investigate whether the aberrant DNA methylation in *GATA3, IL-4* and *TGF-β* is associated with their mRNA expression, we measured the mRNA expression of *GATA3, IL-4* and *TGF-β* in CD4^+^T cells. We found that the mRNA expression of *GATA3* and *TGF-β* was significantly reduced in active BD patients compared to normal subjects (P=0.011, P=0.016; Figure 2A and 2B). However, the mRNA expression of *IL-4* was not significantly different between the two groups (data not shown). We subsequently analyzed the correlation between the DNA methylation and mRNA expression with the Pearson correlation test and found that the methylation level of the CG-7.8.9 units in *GATA3*, CG-2.3.4.5 and CG-10.11 units in *TGF-β* were negatively correlated with their corresponding mRNA expression (P =0.192, r =-0.45; P =0.017, r =-0.762; P =0.169, r =-0.502; respectively. Figure 3A-3C).

### Down-regulated methylation level of *GATA3* and *TGF-β* promoters was detected in CD4^+^T cells from inactive BD patients

**Figure 4:**
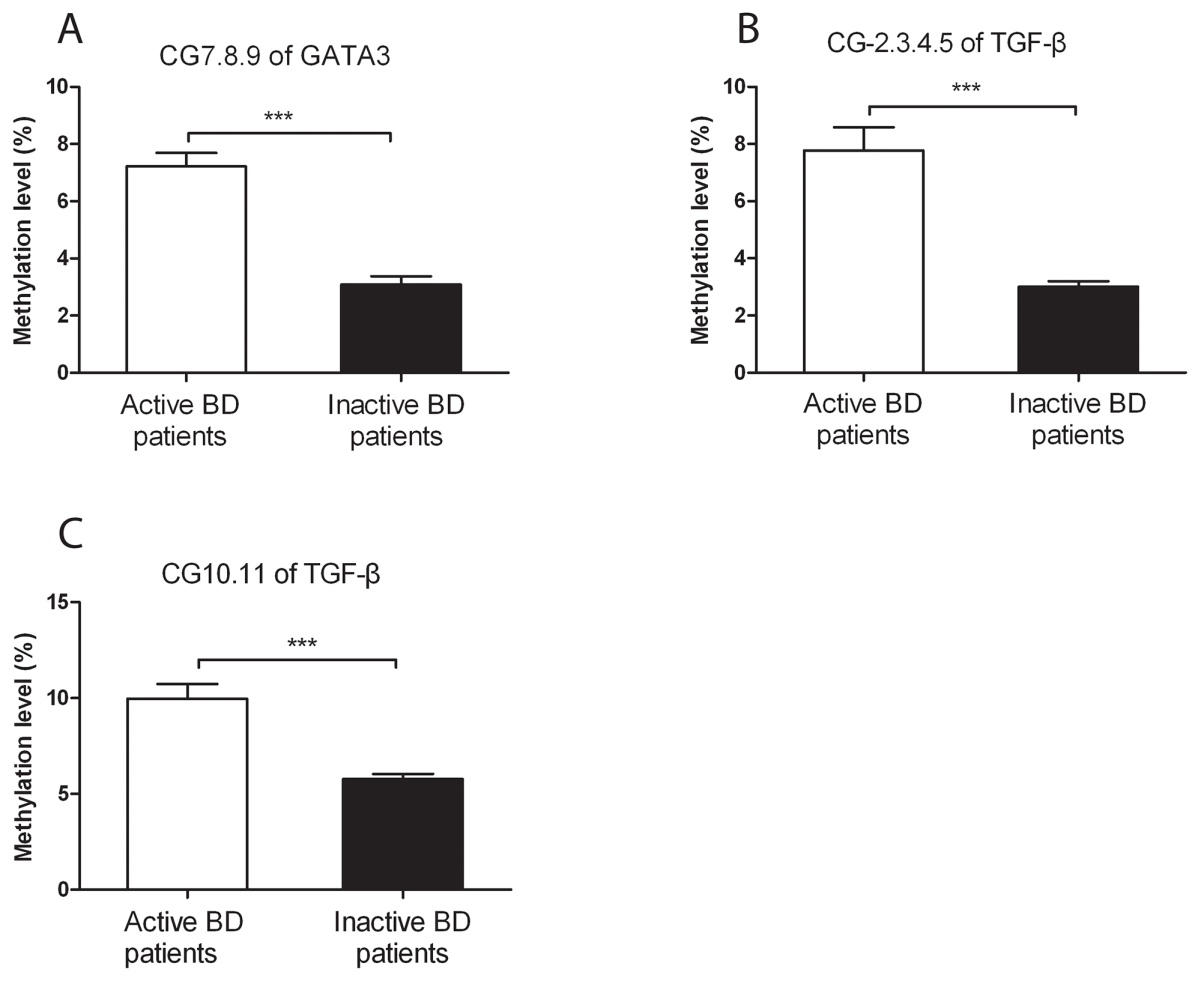
The DNA methylation levels of *GATA3* and *TGF-β* in CD4^+^ T cells from active and inactive BD patients Methylation levels of the CpG-7. 8.9 unit of *GATA3* **(A)** as well as the CpG-2.3.4.5 **(B)** and CpG-10.11 **(C)** of *TGF-β* were all significantly reduced in inactive BD as compared to that observed in active patients. Active BD: n=16; inactive BD: n=11. Data represent mean ± SEM. *** P < 0.001.

To investigate the association between *GATA3* and *TGF-β* promoter methylation with disease activity, we also measured the methylation level of *GATA3* and *TGF-β* in CD4^+^T cells from inactive BD patients. The methylation status of CG-7.8.9 units in *GATA3* was lower in inactive BD than that seen in active patients (P=1.07×10^−6^, Figure 4A). Similarly, compared to active BD patients, the methylation level of CG-2.3.4.5 and CG-10.11 units in *TGF-β* was also significantly reduced in CD4^+^T cells from inactive BD patients (P=5.63×10^−5^, P=1.88×10^−4^, Figure 4B and 4C).

## DISCUSSION

In the present study, we found that the methylation level of the CG-7.8.9 unit of *GATA3*, CG-2 site of *IL-4* as well as CG-2.3.4.5 and CG-10.11 units of *TGF-β* was significantly elevated in CD4^+^T cells from active BD patients. We furthermore showed that the mRNA expression of *GATA3* and *TGF-β* was down-regulated in active BD patients. In addition, the methylation status of CG-7.8.9 unit of *GATA3*, CG-2.3.4.5 and CG-10.11 units of *TGF-β* was markedly lower in inactive BD patients (after treatment with corticosteroids and CsA) than that observed in active BD patients.

The findings from our study are in agreement with earlier observations showing that genetic polymorphisms and copy number variants in many immune-related genes show an association with BD, including the transcription factors and cytokines of CD4^+^T cell subsets [22, 23]. Since genetic predisposition cannot completely explain the development of BD, we have now extended these findings and provide evidence for a possible role of epigenetic regulation of T cell transcription factors in the pathogenesis of this disease.

*GATA binding protein 3* is a very important transcription factor in regulating the differentiation of T helper cells and the expression of Th2 cytokines [24] [25, 26]. Early study had indicated that ablation of *GATA3* resulted in an increased DNA methylation of the *IL-4* gene locus and decreased Th2 cytokines production [24]. Tobacco smoking has been shown to cause a hypomethylation of three CpG sites within *GATA3* and has been suggested to be associated with lung cancer [27]. Another study confirmed that the *GATA3* gene was more strongly methylated in clear cell renal carcinoma (ccRCC) and was partly due to a loss of the expression of this gene [28]. Methylation of the *IL-4* gene was moderately to highly elevated in prostate cancer cells and increased DNA methylation of *GATA3* was observed in androgen negative prostate as compared to androgen positive cells [29]. Hypermethylation is not restricted to cancer since recent studies demonstrated that the *GATA3* gene was also hypermethylated in ulcerative colitis [30]. The *IL-4* gene was shown to be hypomethylated in a mouse model of childhood allergic asthma [31]. In the study reported here, we found that the methylation level of *GATA3* was markedly increased in active BD patients and was negatively associated with gene expression. These data support a role for an abnormal DNA methylation of *GATA3* in the development of BD.

*Transforming growth factor-β* is an important pleiotropic cytokine which participates in the regulation of mammalian development, homeostasis, and the differentiation of Th17 and Treg lymphocytes as well as the pathogenesis of various cancers [32–34]. The microenvironment of mature T lymphocytes determines the cell outcome [35]. *TGF-β* can induce inflammation by promoting the development of Th17 and can inhibit the immune response by facilitating the development of Tregs, thereby suppressing T helper cells [36]. A previous study identified 378 candidate methylated genes in ovarian cancer and reported many of these genes to be relevant in the suppression of *TGF-β* pathway activity [37]. Transforming growth factor-beta-inducible gene h3 (*TGFBI*) hypermethylation has been shown to be associated with paclitaxel-resistance in ovarian cancer and was correlated with the loss of *TGFBI* mRNA expression [38]. Recent studies have demonstrated that the promoter methylation level of *TGF-β*1 was significantly increased in gastric cancer patients, and it was more closely associated with *Helicobacter pylori (H.pylori)* positive patients [39]. Consistent with these previous findings, we found that the methylation level of the *TGF-β* promoter was significantly elevated in active BD patients and was negatively correlated with its mRNA expression.

In a recent study in another autoimmune uveitis entity named Vogt-Koyanagi-Harada (VKH) disease, we also found a hypermethylation of the *GATA3*, *IL-4* and *TGF-β* promoters with a small difference [40]. It is well known that BD and VKH disease are quite different in clinical manifestations. Similar results about hypermethylation of these gene promoters may suggest that both diseases may have similar epigenetic regulation mechanisms. Further studies should be performed to explore whether the abnormal DNA methylation of *GATA3*, *IL-4* and *TGF-β* is also correlated with other uveitis entities. An epigenetic-wide association study (EWAS) found 125 differentially methylated CpG sites in 62 genes that regulate cytoskeletal remodeling in CD4^+^T cell from BD patients [41], but this study did not include *GATA3*, *IL-4* and *TGF-β* genes. The discrepancy may be caused by the fact that this latter EWAS study used the Illumina HumanMethylation450 DNA Analysis BeadChip array. To validate our results and analyze the relationship between methylation changes and clinical phenotypes of BD, further studies will be performed in a large case-control study.

In conclusion, the obtained results suggest that hypermethylation of *GATA3* and *TGF-β* may result in gene transcriptional silencing, which may play a role in the development of BD. Corticosteroids and CsA are the traditional drugs used for the treatment of uveitis, and a part of their beneficial effects may be caused by their ability to restore the aberrant methylation status of *GATA3* and *TGF-β*. Further study is warranted to investigate whether the methylation status of *GATA3* and *TGF-β* promoters may represent potential biomarkers for disease diagnosis or treatment.

## MATERIALS AND METHODS

### Study population

A total of 16 active BD patients (mean age 34.63 ± 6.98 years; 14 males, 2 females) without treatment, 11 inactive BD patients with treatment (mean age 39.55 ± 7.54 years; 10 males, 1 females) and 18 sex- and age-matched healthy subjects (mean age 37.61± 13.63 years; 14 males, 4 females) were recruited from the First Affiliated Hospital of Chongqing Medical University (Chongqing, China) from April 2015 to February 2017, to participate in this study. All participants were Chinese Han. The BD patients were diagnosed by the criteria of the International Study Group for Behcet's disease and all had uveitis [42]. Every patient and healthy individual provided informed consent for the current study. All procedures were in agreement with the tenets of the Declaration of Helsinki and approved by Ethics Research Committee of Chongqing Medical University.

### CD4^+^T-cell isolation

Venous blood was drawn from participants using heparin-coated syringes. Peripheral blood mononuclear cells (PBMCs) were separated from blood samples by Ficoll-Hypaque density gradient centrifugation. CD4^+^T cells were purified from PBMCs using CD4 mAb-conjugated magnetic microbeads (Miltenyi Biotec, Bergisch Gladbach, Germany) in accordance with the manufacturer's protocol. The quality and purity of obtained CD4^+^T cells were measured by flow cytometry and determined to be > 95%.

### DNA extraction

Genomic DNA was extracted from isolated CD4^+^T cells using the QIAamp DNA Blood Mini Kit (Qiagen, Valencia, California, USA) according to the manufacturer's instruction. The DNA concentration and absorbance ratios of each sample were detected by a Nanodrop 2000 apparatus (Thermo Fisher Scientific, Wilmington, DE, USA).

### Sodium bisulfite modification

Genomic DNA (1 μg) of every sample was treated with the EZ DNA Methylation Kit (Agena Bioscience, California, USA) according to the instruction manual. The polymerase chain reaction (PCR) was performed during the bisulfate conversion, which included 20 cycles of the following two steps: 95°C for 30 seconds, 50°C for 15 minutes.

### Primer design for EpiTYPER assay

DNA sequences and CpG units of gene promoters were determined by the UCSC website (http://www.genome.ucsc.edu). According to the identified DNA sequence, the upstream and downstream primers were designed by the EpiDesigner online application (http://www.epidesigner.com). For each pair of primers, an additional T7 promoter tag and a 10-mer tag were added to the reverse and forward primer respectively. The CpG specific primer sequences and detailed information of the target genes are shown in Table 1.

### DNA methylation analysis by MassARRAY

Quantitative methylation analysis was performed by the Sequenom's MassARRAY system (Sequenom Inc, San Diego, California, USA), which employs matrix-assisted laser desorption/ionization-time of-flight mass spectrometer (MALDI-TOF-MS). Bisulfite-treated DNA was then subjected to the following reactions: PCR amplification (94°C for 4 min; 45 cycles of 94°C for 20s, 56°C for 30 s, and 72°C for 1 min; then 72°C for 3 min); dephosphorylated by shrimp alkaline phosphatase (SAP) (Sequenom) (37°C for 20min, 85°C for 5min); followed by base specific cleavage (37°C for 3h). The resultant products were purified by the clean resin (Sequenom) and spotted on a 384-element silicon chip (SpectroCHIP, Sequenom, USA). Mass spectra were obtained using MassARRAY mass spectrometer and analyzed using EpiTYPER software v1.2 (Sequenom Inc, San Diego, California, USA) and the results were shown as percentages of methylation of each CpG site. The results were normalized by the methylation level of the test standard within the methylation analysis kit (Sequenom, Inc., San Diego, USA). Each sample was subjected to duplicate independent analyses and poor-quality and non-applicable data were eliminated in the calculations.

### RNA extraction and real-time PCR

Total RNA was extracted from CD4^+^T cells by TRIzol reagent (Invitrogen, CA, USA) then used to synthesize complementary DNA by the PrimeScript RT kit (Takara, Dalian, China). Real-time PCR was performed by SYBR Premix (Takara Biotechnology, Dalian, China) with the ABI Prism 7500 system (Applied Biosystems, CA, USA). The mRNA expression was analyzed by the 2^−ΔΔCt^ method. The primer sequences of β-actin and target genes for PCR were as follow: β-actin: forward: 5′-GGATGCAGAAGGAGATCACTG-3′, reverse: 5′-CGATCCACACGGAGTACTTG-3′; *GATA3*: forward: 5′-GCGGGCTCTATCACAAAATGA-3′, reverse: 5′-GCTCTCCTGGCTGCAGACAGC-3′; *IL-4*: forward: 5′-CACAACTGAGAAGGAAACCTTCTG-3′, reverse: 5′-CTCTCTCATGATCGTCTTTAGCCTTTC-3′; *TGF-β*: forward: 5′-GGACACCAACTATTGCTTCAG-3′, reverse: 5′-TCCAGGCTCCAAATGTAGG-3′.

### Statistical analysis

The statistical analyses were performed using SPSS 17.0 software (SPSS Inc, Chicago, Illinois, USA) and GraphPad Prism 5 software (GraphPad Software, Inc., CA). The Mann-Whitney test and independent-sample t-test were used to analyze the methylation level and mRNA expression, and the Pearson correlation test was used for the analysis of the correlation between DNA methylation and mRNA expression. A *p*-value less than 0.05 was regarded as significant difference.

## Abbreviations

BD: Behcet's disease
ccRCC: clear cell renal carcinoma
CsA: Cyclosporin A
EWAS: epigenome-wide association studies
FOXP3: forkhead box protein 3
GATA3: GATA binding protein 3
H.pylori: Helicobacter pylori
IFN-γ: interferon-γ
IL: interleukin
MALDI-TOF-MS: matrix-assisted laser desorption/ionization-time of-flight mass spectrometer
PBMCs: peripheral blood mononuclear cells
PCR: polymerase chain reaction
RORγt: retinoid-related orphan nuclear receptor γt
SAP: shrimp alkaline phosphatase
TBX21: T-box transcription factor 21
TGF-β: transforming growth factor-β
TGFBI: transforming growth factor-beta-inducible gene h3
Th: T helper
Treg: regulatory T cell
VKH: Vogt-Koyanagi-Harada
